# Supplementary material for: Tead1a Initiates Transcriptional Priming Through the TEAD1a/YAP‐Notch1‐Spi1/Cebpα Axis to Promote Neutrophil Fate
Source: Adv Sci (Weinh). 2025 Aug 28;12(41):e05441. doi: 10.1002/advs.202505441 (PMC12591127; doi:10.1002/advs.202505441)
Supplement: Supplementary file 4 — Supplementary Dataset [file ADVS-12-e05441-s004.zip › 3-6.zip!/3-6/02-6.cluster/cluster15/Enrich/ego_CC.pdf]

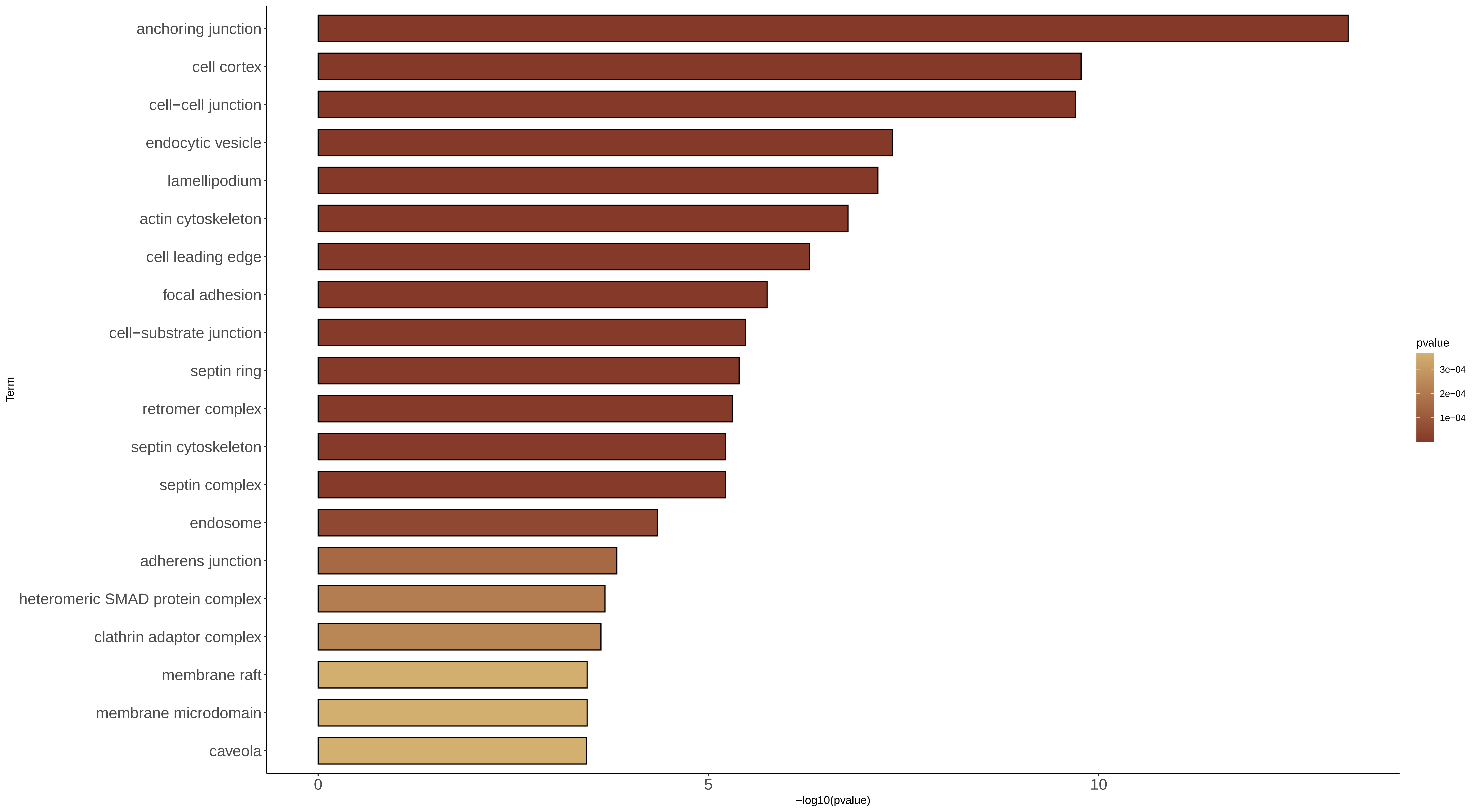
anchoring junction
cell cortex
cell−cell junction
endocytic vesicle
lamellipodium
actin cytoskeleton
cell leading edge
focal adhesion
cell−substrate junction
septin ring
retromer complex
septin cytoskeleton
septin complex
endosome
adherens junction
heteromeric SMAD protein complex
clathrin adaptor complex
membrane raft
membrane microdomain
caveola
Term
0
5
10
−log10(pvalue)
pvalue
3e−04
2e−04
1e−04
